# How Do Maternal Subclinical Symptoms Influence Infant Motor Development during the First Year of Life?

**DOI:** 10.3389/fpsyg.2016.01685

**Published:** 2016-11-01

**Authors:** Giulia Piallini, Stefania Brunoro, Chiara Fenocchio, Costanza Marini, Alessandra Simonelli, Marina Biancotto, Stefania Zoia

**Affiliations:** ^1^Department of Developmental Psychology and Socialization, University of PaduaPadua, Italy; ^2^Struttura Complessa Tutela Salute Bambini Adolescenti Donne Famiglia, Azienda Sanitaria Universitaria Integrata di TriesteTrieste, Italy

**Keywords:** maternal depressive symptomatology, infant motor development, PDMS-2, SCL-90-R, fine motor abilities, gross motor abilities

## Abstract

An unavoidable reciprocal influence characterizes the mother-child dyad. Within this relationship, the presence of depression, somatization, hostility, paranoid ideation, and interpersonal sensitivity symptoms at a subclinical level and their possible input on infant motor competences has not been yet considered. Bearing in mind that motor abilities represent not only an indicator of the infant's health-status, but also the principal field to infer his/her needs, feelings and intentions, in this study the quality of infants' movements were assessed and analyzed in relationship with the maternal attitudes. The aim of this research was to investigate if/how maternal symptomatology may pilot infant's motor development during his/her first year of life by observing the characteristics of motor development in infants aged 0–11 months. Participants included 123 mothers and their infants (0–11 months-old). Mothers' symptomatology was screened with the Symptom Checklist-90-Revised (SCL-90-R), while infants were tested with the Peabody Developmental Motor Scale-Second Edition. All dyads belonged to a non-clinical population, however, on the basis of SCL-90-R scores, the mothers' sample was divided into two groups: normative and subclinical. Descriptive, *t*-test, correlational analysis between PDMS-2 scores and SCL-90-R results are reported, as well as regression models results. Both positive and negative correlations were found between maternal perceived symptomatology, *Somatization* (SOM), *Interpersonal Sensitivity* (IS), *Depression* (DEP), *Hostility* (HOS), and *Paranoid Ideation* (PAR) and infants' motor abilities. These results were further verified by applying regression models to predict the infant's motor outcomes on the basis of babies' age and maternal status. The presence of positive symptoms in the SCL-90-R questionnaire (subclinical group) predicted good visual-motor integration and stationary competences in the babies. In particular, depressive and hostility feelings in mothers seemed to induce an infant motor behavior characterized by a major control of the environmental space. When mothers perceived a higher level of hostility and somatization, their babies showed difficulties in sharing action space, such as required in the development of stationary positions and grasping abilities. In a completely different way, when infants can rely on a mother with low-perceived symptoms (normative group) his/her motor performances develop with a higher degree of freedom/independence. These findings suggest, for the first time, that even in a non-clinical sample, mother's perceived-symptoms can produce important consequences not in infant motor development as a whole, but in some specific areas, contributing to shape the infant's motor ability and his/her capability to act in the world.

## Introduction

The birth of a child entails a total restructuration of the identity of the new parents, as well as the emergence of the new role of parenthood within the couple. In fact, during pregnancy the process of transition to parenthood emerges exclusively on an imaginative level as the couple begins to compare the idea of becoming parents and co-parents. However, the concrete access to parenthood takes place at the time of baby's birth (Von Klitzing et al., 1999; Carneiro et al., 2006; Simonelli et al., 2012). Given the amount and depth of changes that occur during pregnancy and the postpartum period, it is not surprising that many women find it difficult to deal with this situation. As much as they develop depressive problems, these may have severe effects not just on the mothers, but also on the children and on the family systems themselves. Many studies show that depression can interfere with parenting skills and with the establishment of an attachment bond (Gotlib et al., 1991). Parents play an essential role in the survival and development of the infant and the dyadic relation between parent and infant represents the first, and most important, interaction for the baby. Appropriate interactions with the caregiver are fundamental for the development of the infant and the structure underlying neural mechanisms responsible for the infant's typical or atypical development. Being a parent and adequately responding to the offspring necessities, requires a huge amount of cognitive resources and self-awareness, allowing parents to implement suitable care-giving behaviors (e.g., to consistently respond or to promote baby's actions). In case of parental psychopathology (e.g., depression, post-partum depression, etc.), these abilities may result substantially compromised and the mother may not be able to meet the child's needs functionally, leading to consequences on the infant's development and wellbeing, as well as on the establishment of a healthy and functional parent-infant relationship. During the first months of the newborns' life, many women present the risk of developing a depressive disorder which can affect maternal responsiveness, leading to short and long term consequences on child development (Milgrom et al., 2004), on maternal functioning and on the early mother-child interactions (Parolin and Sudati, 2014).

Postpartum depression negatively affects woman's ability to take on the maternal role and can lead to difficulties in caregiving toward the child, feelings of guilt and poor self-efficacy (Monti and Agostini, 2006). As a consequence, an impoverishment of the dyadic mother-child relationship occurs, since a depressed mother may not be tuned to the child's needs and may be unable to respond appropriately (Parolin and Sudati, 2014). The higher risks facing the child consist in the impairment of cognitive and motor development, poor ability of self-regulation, low self-esteem and behavioral problems (Goodman and Gotlib, 1999; Field, 2010).

In a study conducted in the Barbados Island, Galler et al. (2000) found significant relationships between maternal moods and infant cognitive development; in particular, they found an association between maternal depression and low motor performance in 6 month old infants, without establishing a causal relationship between these factors. Maternal moods at 6 months were associated with lower scores in motor development of the children at the same age; in particular, the children of mothers who reported less confidence, greater hopelessness and lower pleasure in being with the baby, obtained lower scores in the evaluation of motor development (Galler et al., 2000). Although no independent relationships emerged between feeding practices and infant cognitive development, the combination of diminished infant feeding intensity and maternal depression predicted delays in the infant's social development. With their findings, Galler and colleagues demonstrated the need to monitor maternal moods during the postpartum period, in order to relate health programs to infant cognitive development. In the same way, using the Bayley Scales (Bayley, 1969), Lyons-Ruth et al. (1986) found that 12 month old children of depressed mothers were more likely to exhibit unstable, avoidant attachment behaviors and slowed development, in particular lower levels of motor development than children of non-depressed mothers (Lyons-Ruth et al., 1986; Murray and Cooper, 1997).

Moreover, Cornish et al. (2005) examined the impact of brief and chronic depression in a normative sample, finding an association between chronic maternal depression and lower infant cognitive and psychomotor development, with the effects being similar for boys and girls; on the contrary, brief depression did not significantly impact infant performance (Cornish et al., 2005). Nasreen et al. (2013), in a study conducted in Bangladesh, found that postpartum depression was associated with impaired child's growth at 2–3 months and in motor development at 6–8 months. Also, the age of the mother, the child's weight in relation to the mother's age and the maternal anxiety about the infant's care, were directly associated with motor development (Nasreen et al., 2013).

It is important to underline that the relationship between postpartum depression and outcomes on child's development is not linear or direct; indeed different variables might play a role, such as genetic influences, educational level of the mother, child's gender, economic status and involvement of the father or other adults (Sohr-Preston and Scaramella, 2006).

Focusing on the infants, their motor development improves with broad inter-individual variability, not just from the temporary point of view, but also in the strategies with which the infant develops a specific motor skill (Touwen, 1976). Developmental progression is generally divided into a sequence of stages, which are not linear and identical in every child (Thelen, 1995; Camaioni and Di Blasio, 2002; Sheridan, 2009). Considering gross-motor abilities, in the first year of life the child gradually learns posture and balance control and later he/she learns to move around through space, while fine-motor skills rely on the child's ability to use his/her visual-perceptive competences to accomplish eye-hand coordination tasks, for example reaching and grasping objects (Folio and Fewell, 2000). Since the first month of life, newborns are able to stare at a slowly-moving stimulus in his/her field of vision, at 15–25 cm of distance; at 3 months he/she shifts his/her gaze from one side to the other and can keep a rattle in his/her hand for a few seconds. However, the reach-to-grasp action remains strongly linked to vision and partially depending on the environmental prompts, mainly provided by the caregiver.

The fundamental stages of motor development represent a useful instrument to assess the timing and extent of a child's progresses. Nevertheless, even if motor development in the first stages appears biologically determined, afterwards it depends on the combination of practice and learning from a continuous two-way interaction between biological and environmental factors (Zoia et al., 2013).

The newborn's first approach to the world takes place in the relationship with his/her caregiver, usually the mother. The infant takes the first clues to build his/her own environment and experiences from the repertoire of facial expressions, tones, gestures and postures of the mother (Stern, 1977). The transition from reflexes to the ability of reach-to-grasp takes place within this framework, and the infant acquires shared attention for an object, mainly proposed by the caregiver, in a close relational space of interaction with it. In the meantime, the infant also starts to develop actions like rolling, creeping and standing that widen his/her action space.

In the present study, we capitalize on the above-mentioned studies to explore the relation between maternal psychological status and motor development during the first year of life. In particular, when mother-infant dyads are characterized by maternal anxiety or depressive symptoms, even without reaching a clinical level, we raise the issue of possible influences on the development of motor experiences and specific motor skills. The development of reflexes mainly depends on a neurophysiological maturation of the central nervous system, while reaching an object, as well as gross-motor acquisitions (like flexing legs, extending arms, pushing up, rolling, crawling, standing), can be influenced by the differences in environmental stimulations.

Our central question is not just if motor development is general related to maternal psychological status, but whether maternal conditions may differently modulate specific subcomponents of motor development (fine-motor versus gross-motor skills). For example, if the mother perceives somatization or depressive symptoms at a subclinical level, could her status influence her baby's motor experiences? If motor abilities can be influenced by maternal psychic status, is it important what kind of psychopathological symptoms the mother perceives?

## Materials and methods

### Participants

We recruited 134 mother-infant dyads from the normative population; 5 were excluded for impossibility to complete the infant motor protocol, 4 were excluded due to the age of the infants (≥12 months); 6 were excluded for incompleteness of the maternal protocol. Overall, we conducted the analysis over 119 dyads.

Mothers were 23–43 years old (mean age = 32.87 years). Eighteen mothers (15.1%) had medical problems during pregnancy and 25 mothers (21%) had problems during delivery; 28 mothers (23.5%) had at least one previous experience of abortion. In our sample, 67 mothers (56.3%) had a high educational level and 49 (41.2%) had a medium or low educational level; 100 mothers (84%) had an occupation and 106 mothers (89.1%) were married or lived together with the baby's father. None were socio-economically disadvantaged. All mothers were happy to take part in the research. They were assessed with the Symptom Checklist-90-Revised questionnaires (SCL-90-R; Derogatis, 1983; Italian version by Sarno et al., 2011) to investigate the perception of psychological symptomatology.

Infants were between 0 and 11 months old (mean age = 5.98 months, 55 boys and 64 girls). None of them encountered problems during pregnancy and they were born at term, ranging between 37 and 40 weeks of gestation, with APGAR score between 7/8 and 9/10 and birthweight between 2.3 and 4.4 kg (mean = 3.3 kg, SD = 0.51). Each baby was assessed with Peabody Developmental Motor Scale-Second Edition (PDMS-2; Folio and Fewell, 2000) to evaluate his/her motor development.

### Procedures and instruments

The entire procedure had been submitted to and approved by the Ethic Committee of the University of Padua and each mother agreed to participate with his/her baby to the study by signing the informed consent.

Dyads were recruited from the Babylab Database of the University of Padua and thanks to the collaboration of some Pediatricians. The procedure took place in a laboratory of the Department of Developmental and Social Psychology of the University of Padua. The entire procedure took two sessions, each lasting about 60 min, during which two psychologists administered the PDMS-2 test to the infant, while, in another room, the mother was interviewed and responded to the SCL-90-R questionnaire in presence of another researcher. In this way, data about offspring, marital status, school level, employment and previous problematic pregnancies or experiences of abortion were collected, as well as information about psychic discomfort.

The *SCL-90-R* (Derogatis, 1983; Sarno et al., 2011) was used to evaluate psychological problems and symptoms of psychopathology. This is a relatively brief self-report questionnaire published by the Clinical Assessment division of the Pearson Assessment & Information group. It is designed to evaluate a broad range of psychological problems and symptoms of psychopathology. It consists of 90 items and takes 12–15 min to administer, yielding nine scores relative to primary symptom dimensions and three scores related to global distress indices. The primary symptom dimensions assessed are: somatization, obsessiveness-compulsiveness, interpersonal sensitivity, depression, anxiety, hostility, phobic anxiety, paranoid ideation, psychoticism, and a category of “additional items” useful to evaluate other symptoms. The three indices are: the Global Severity Index (GSI), considered to be a sensitive single quantitative indicator, concerning the respondent's psychological distress status; the Positive Symptom Distress Index (PSDI), considered to be an intensity measure which may also provide information about respondent's distress style; the Positive Symptom Total (PST) which reveals the number of symptoms that the respondent has endorsed to any degree. Particularly, criteria to interpret the GSI score are: with *T* < 55 subjects' general level reported is normative, 55 ≤ *T* < 65 subject reports from moderate to high level of disease; *T* ≥ 65 subject reports a level of disease over the clinical cut-off. Criteria to interpret PST and PSDI are identical: for the PSDI, *T* < 55 indicates that the intensity of the symptoms reported by the subject is normative, 45 ≤ *T* < 65 underlines a moderate/high level of intensity of the symptoms, and *T* ≥ 65 the intensity of the symptoms reported by the subject overcomes the clinical cut-off; regarding the PST, *T* < 55 means that the number of symptoms reported by the subject is normative, 45 ≤ *T* < 65 subject reports a moderate/high number of symptoms, and *T* ≥ 65 the number of symptoms reported by the subject exceeds the clinical cut-off.

The PDMS-2 is a test designed to assess for motor development in children aged from 0 months to 5 years and 11 months. It is composed of 6 subtests: *Reflexes* (administered only from 0 to 11 months), *Stationary, Locomotion, Object Manipulation* (administered to children aged 12 months and older)—that compose the *Gross Motor Quotient* (GMQ)—*Grasping* and *Visual-Motor Integration*—that compose the Fine *Motor Quotient* (FMQ). The best estimate of child's motor abilities is the *Total Motor Quotient* (TMQ), a combination of the results of Gross and Fine Motor subtests (Folio and Fewell, 2000). According to the Italian standardization of the PDMS-2, all the babies included in this study showed a *TMQ* within the normal range (between 84 and 113).

### Descriptive statistical analysis

The internal consistence reliability of SCL-90-R questionnaire and PDMS-2 test was evaluated by computing Cronbach's α coefficient. For the SCL-90-R questionnaire, the Cronbach's Alpha coefficients are included between 0.706 (ANX) and 0.831 (DEP). Phobic Anxiety (PHOB) and Psychoticism (PSY) subscales have not been considered in the analysis because they resulted unreliable (Cronbach's Alpha = 0.576 and 0.608 respectively); while for the PDMS-2 sub-scales, the Cronbach's Alpha coefficients are included between 0.877 (Reflexes) and 0.965 (Visual-Motor Integration). In the same way, the coefficients calculated on each Motor Quotient present great levels of reliability (GMQ = 0.978; FMQ = 0.977).

Therefore, descriptive statistics were applied to all variables (see below).

### Data analysis strategies

Based on the results of the descriptive analysis (reported in the section below) the entire group of mothers was divided into 2 samples according to their perceived psychological symptomatology: we used as a criterion the scores *T* ≥ 55 in the global indices of the SCL-90-R (GSI, PST, or PSDI). In this way we obtained two groups:

Normative group (*N* = 72). Mothers of this sample obtained subclinical/clinical score in none of the global indices GSI, PST, and PSDI of the SCL-90-R.

Subclinical group (*N* = 47). Mothers of this sample obtained subclinical/clinical score in at least one of the global indices GSI, PST and PSDI of the SCL-90-R.

This methodological choice allowed to identify those mothers with some kind of psychological symptomatology, remindful that none of these mothers presented a psychopathological diagnosis.

The *t*-test over these groups was calculated. Furthermore, correlations between maternal scores in SCL-90-R questionnaire and children's PDMS-2 scores were analyzed and regression models to predict infant motor outcomes on the basis of babies' age and maternal status were applied.

## Result

### Descriptive results

In Table 1 a description of the mothers group examined with the SCL-90-R is reported. With respect to the SCL-90-R subscales, a total of nine mothers scored above *T* = 55 in the Global Severity Index (i.e., 8 mothers reached a GSI score within the subclinical range and 1 mother scored higher than *T* = 65, which represents the cut-off for the clinical range; Gibson and Pick, 2000; Adolph and Berger, 2005, 2006). Twelve mothers topped cut-off *T* = 55 in the Positive Symptom Total (only 1 mother scored PST with *T* ≥ 65), while thirty-nine mothers surpassed *T* = 55 in the Positive Symptom Distress Index (13 mothers over 39 scored PSDI with *T* ≥ 65). The results of each SCL-90-R scale are detailed in the table below.

**Table 1:** **Descriptive results: SCL-90-R**.

	***N***	**Min**	**Max**	**Mean**	**SD**	***T* < 55**	**55 ≤ *T* < 65**	***T* ≥ 65**
SOM	119	37	75	44.76	6.818	109	8	2
OC	119	36	75	46.04	7.508	106	10	3
IS	119	37	70	45.46	6.857	106	11	2
DEP	119	37	73	46.24	6.8	105	12	2
ANX	119	39	64	44.49	4.984	113	6	0
HOS	119	40	75	47.18	7.799	94	22	3
PAR	119	37	68	43.57	7.174	107	11	1
GSI	119	36	65	44.93	5.897	110	8	1
PST	119	28	66	43.09	7.859	107	11	1
PSDI	119	34	75	51.3	9.152	80	26	13

Focusing on the PDMS-2 test, the descriptive results of each subscale and quotient split for boys and girls were reported in Table 2. The subscales mean scores for the whole sample were: *Reflexes* = 7.29 (*SD* = 4.285), *Stationary* = 24.56 (*SD* = 9.084), *Locomotion* = 25.91 (*SD* = 17.004), *Grasping* = 23.2 (*SD* = 13.269), *Visual-Motor Integration* = 29.77 (*SD* = 17.909). The quotients for the whole sample were: *GMQ* = 57.76 (*SD* = 29.286), *FMQ* = 52.97 (*SD* = 30.115) and *TMQ* = 110.74 (*SD* = 58.885).

**Table 2:** **Descriptive results divided according to the gender: PDMS-2**.

**Gender**		**Min**	**Max**	**Mean**	**SD**	**Variance**
Males *N* = 55	REFLEXES	1	15	7.24	4.36	18.99
	STATIONARY	4	38	23.80	9.51	90.42
	LOCOMOTION	6	71	25.84	18.07	326.47
	GRASPING	2	42	22.18	12.91	166.78
	VISUAL-MOTOR INTEGRATION	4	70	30.53	18.59	345.70
	GMQ	12	122	56.87	30.867	952.78
	FMQ	7	108	52.71	30.924	956.32
	TMQ	19	229	109.58	61.289	3756.40
Females *N* = 64	REFLEXES	1	15	7.34	4.255	18.10
	STATIONARY	8	38	25.22	8.724	76.11
	LOCOMOTION	2	65	25.97	16.179	261.75
	GRASPING	3	49	24.08	13.606	185.12
	VISUAL-MOTOR INTEGRATION	5	69	29.13	17.421	303.48
	GMQ	14	114	58.53	28.080	788.51
	FMQ	8	110	53.20	29.645	878.80
	TMQ	22	224	111.73	57.206	3272.58

### *T*-test

The *t*-test analysis on mothers who belonged to the normative and subclinical sample were conducted to investigate if any differences emerged in the motor development of those children whose mothers showed sub-clinical scores in SCL-90-R compared to the normative group. Furthermore, comparisons considering the infants' gender (difference between boys and girls in the entire sample, as well as boys vs. girls in the normative and in the subclinical sample, and finally, normative vs. subclinical sample in the boys' and girls' scores) were also examined. No significant results were found: *t*-test showed no differences regardless of the children's age as the mothers belonged to the normative or subclinical group (Table 3A). In the same way, no differences were found in the PDMS-2 scores according to infants' gender (Table 3B). This is consistent with the PDMS-2 American and Italian standardizations, which revealed no significant differences in the test performances between boys and girls (Folio and Fewell, 2000; Biancotto et al., 2016).

**Table 3A:** ***T*-test: comparison between Subclinical and Normative group**.

	**Subclinical**			**Normative**			***t*****-test**		
	**Mean**	**SD**	***N***	**Mean**	**SD**	***N***	***t***	***df***	***p***
**GROUP 0–6 MONTHS**									
REFLEXES	4.60	2.697	40	4.00	2.204	29	0.97	67	0.336
STATIONARY	18.55	6.488	40	17.28	5.133	29	0.86	67	0.391
LOCOMOTION	14.98	8.263	40	13.00	6.251	29	1.07	67	0.290
GRASPING	14.20	9.002	40	12.76	7.949	29	0.68	67	0.499
V-M INT.	18.00	8.715	40	16.59	7.149	29	0.71	67	0.483
GMQ	38.13	16.185	40	34.28	12.300	29	1.06	67	0.293
FMQ	32.20	17.350	40	29.34	14.296	29	0.71	67	0.477
TMQ	70.33	32.827	40	63.62	25.873	29	0.90	67	0.371
**GROUP 7–11 MONTHS**									
REFLEXES	11.44	2.539	32	11.22	2.602	18	0.28	48	0.779
STATIONARY	33.53	2.423	32	33.72	2.492	18	−0.26	48	0.799
LOCOMOTION	40.78	12.130	32	44.56	12.949	18	−1.01	48	0.317
GRASPING	36.78	4.241	32	35.89	2.246	18	0.81	48	0.421
V-M INT.	46.25	14.366	32	47.89	10.493	18	−0.42	48	0.679
GMQ	85.75	15.419	32	89.50	16.745	18	−0.78	48	0.437
FMQ	83.03	15.818	32	83.78	11.755	18	−0.17	48	0.864
TMQ	168.78	29.726	32	173.28	27.729	18	−0.52	48	0.609

**Table 3B:** ***T*-test: comparison between Males and Females**.

**Gender**	**Males**			**Females**			***t*****-test**		
	**Mean**	**SD**	***N***	**Mean**	**SD**	***N***	***t***	***df***	***p***
REFLEXES	7.24	4.36	55	7.34	4.255	64	−0.135	117	0.893
STATIONARY	23.8	9.51	55	25.22	8.724	64	−0.841	117	0.402
LOCOMOTION	25.84	18.07	55	25.97	16.179	64	−0.042	117	0.967
GRASPING	22.18	12.91	55	24.08	13.606	64	−0.769	117	0.443
V-M INT.	30.53	18.59	55	29.13	17.421	64	0.421	117	0.675
GMQ	56.87	30.867	55	58.53	28.08	64	−0.304	117	0.762
FMQ	52.71	30.924	55	53.2	29.645	64	−0.088	117	0.930
TMQ	109.58	61.289	55	111.73	57.206	64	−0.196	117	0.845

### Correlational results

The correlations between the scores obtained by the infants at the PDMS-2 subscales and those of the mothers at the SCL-90-R questionnaire were calculated. Due to the PDMS-2 structure, the scores in the test increase with infants' age; for this reason we calculated partial correlations considering the infant's age as a control variable.

All the results found between the maternal scores and the infants' PDMS-2 scores were reported in Table 4, divided into the two groups of mothers (normative vs. sub-clinical).

**Table 4:** **Correlational results in Normative and Sub-clinical group**.

	**SOM**	**OC**	**IS**	**DEP**	**ANX**	**HOS**	**PSR**	**GSI**	**PST**	**PSDI**
**NORMATIVE GROUP df** = **69**										
REFLEXES	0.091	0.096	−0.030	0.076	−0.035	0.016	−0.056	0.037	0.055	−0.052
STATIONARY	−0.102	−0.117	−0.033	−0.164	−0.122	−0.148	0.000	−0.162	−0.149	−0.059
LOCOMOTION	0.004	0.051	0.236^*^	0.101	0.000	0.138	0.217	0.147	0.093	0.126
GRASPING	−0.115	−0.019	−0.072	−0.136	−0.176	−0.168	−0.035	−0.149	−0.166	−0.045
V-M INT.	−0.188	−0.147	0.002	−0.122	−0.134	−0.202	−0.112	−0.142	−0.164	−0.164
GMQ	−0.011	0.020	0.155	0.035	−0.055	0.052	0.146	0.059	0.029	0.058
FMQ	−0.217	−0.128	−0.040	−0.176	−0.209	−0.258^*^	−0.109	−0.200	−0.227	−0.157
TMQ	−0.134	−0.065	0.059	−0.086	−0.153	−0.125	0.013	−0.087	−0.119	−0.062
**SUB-CLINICAL GROUP df** = **44**										
REFLEXES	−0.315^*^	0.110	−0.028	0.162	0.081	0.130	0.212	0.056	−0.007	0.050
STATIONARY	−0.086	0.087	0.214	0.387^*^	0.218	0.088	0.328^*^	0.215	0.157	0.029
LOCOMOTION	−0.065	0.135	0.403^*^	0.301^*^	0.147	0.374^*^	0.439^*^	0.321^*^	0.276	0.069
GRASPING	−0.078	0.198	0.164	0.282	0.122	0.045	0.339^*^	0.191	0.145	−0.059
V-M INT.	−0.249	0.038	0.169	0.344^*^	0.142	0.460^**^	0.253	0.194	0.074	0.090
GMQ	−0.138	0.153	0.372^*^	0.382^*^	0.195	0.343^*^	0.482^**^	0.325^*^	0.260	0.072
FMQ	−0.214	0.146	0.212	0.401^*^	0.169	0.334^*^	0.375^*^	0.245	0.138	0.024
TMQ	−0.189	0.163	0.324^*^	0.426^*^	0.199	0.370^*^	0.471^**^	0.314^*^	0.221	0.054

* *p < 0.05*,

** *p < 0.001, p > 0.06*.

In the group of normative mothers, the Interpersonal Sensitivity (IS) subscale of SCL-90-R questionnaire was positive correlated only with the Locomotion subscale of the PDMS-2 (0.236, *p* < 0.05), therefore locomotion increased with IS. A unique negative correlation was found between the hostility subscale (HOS) of SCL-90-R questionnaire and the Fine Motor Quotient (FMQ; −0.258, *p* < 0.05), which indicated that when mothers perceived low hostility feelings their babies' FMQ score increased.

On the contrary, for the group of subclinical mothers several correlations were found between the maternal psychic status and their babies' motor abilities. More precisely, a unique negative correlation was found between the Somatization (SOM) scale of the SCL-90-R questionnaire and the Reflexes scale of the PDMS-2 (−0.258, *p* = 0.03), that is, a higher presence of somatization symptoms was related with a lower score on baby's reflexes. Diversely, the IS subscale of the SCL-90-R questionnaire positively correlated with the Locomotion, Gross-Motor Quotient (GMQ) and Total Motor Quotient (TMQ) subscale of the PDMS-2 test. The presence of depression symptoms (DEP subscale of SCL-90-R questionnaire) showed positive correlations with the Stationary, Locomotion, Visual-motor integration, GMQ, FMQ, and TMQ of the PDMS-2 (see Table 3 for values). The HOS scale of the SCL-90-R revealed positive correlations with Locomotion, Visual-motor integration, GMQ, FMQ, TMQ, and Grasping subscale of PDMS-2; similarly the paranoid ideation scale (PAR) correlated with these PDMS-2 subscales, plus the Stationary subscale. Lastly, the Global Severity Index (GSI of the SCL-90-R) showed a positive correlation with Locomotion, GMQ and TMQ scores of the PDMS-2 (see Table 3 for values).

### Regression models results

Regression models analyses were performed for a deeper comprehension of the findings described in the above section. Considering each group of mothers separately (normal and subclinical), the five psychopathological subscales of the SCL-90-R questionnaire that revealed some correlations with the PDMS-2 subscales were considered as predictive factors of the infants' motor abilities. Therefore, somatization, interpersonal sensitivity, depression, hostility and paranoia SCL-90-R subscales, along with the infants' age, were regarded as possible predictors of the motor competence in the different subscales measured by the PDMS-2. Regression analyses were conducted for each dependent motor variable: reflexes, stationary, locomotion, grasping, visual-motor integration subtests and the total motor quotient.

Overall, infants' age had a significant and positive effect on all the PDMS-2 subscales and on the total motor quotient. In particular, for the normative group of mothers the regression models always resulted significant: Total Motor Quotient [*F*_(6, 67)_ = 146,051; *p* < 0.001], Reflexes [*F*_(6, 67)_ = 30,168; *p* < 0.001]; Stationary [*F*_(6, 67)_ = 85,614; *p* < 0.001], Locomotion [*F*_(6, 67)_ = 67,745; *p* < 0.001], Grasping [*F*_(6, 67)_ = 59,495; *p* < 0.001] and Visual-motor Integration [*F*_(6, 67)_ = 63,103; *p* < 0.001], with infants' age as the only significant predictor (see Table 5 for Beta coefficients). Infants' age was a significant predictor also for the subclinical group. However, in this group of mothers some perceived symptoms seem to influence the infants' motor competence and the way in which they act in the environment.

**Table 5:** **Regression models results**.

	**TMQ**		**REFLEXES**		**STATIONARY**		**LOCOMOTION**		**GRASPING**		**V-M INT**	
	**Subcl.G**	**(N.G.)**	**Subcl.G**	**(N.G.)**	**Subcl.G**	**(N.G.)**	**Subcl.G**	**(N.G.)**	**Subcl.G**	**(N.G.)**	**Subcl.G**	**(N.G.)**
**PREDICTORS**												
Age in months	0.96^**^	(0.97)^**^	0.88^**^	(0.86)^**^	0.95^**^	0.95^**^	0.93^**^	0.88^**^	0.91^**^	0.94^**^	0.95^**^	0.93^**^
SOM	−0.08^*^	(−0.02)	−0.15^*^	0.06	−0.08	0.02	−0.05	−0.03	−0.08	−0.002	−0.08	−0.06
IS	−0.01	(0.04)	−0.15	−0.03	−0.008	0.005	0.07	0.03	−0.04	−0.009	−0.04	0.12
DEP	0.09	(−0.03)	−0.16	0.05	0.16^**^	−0.08	0.02	−0.03	0.13	−0.04	0.06	−0.013
HOS	−0.01	(0.02)	−0.05	−0.03	−0.13^*^	−0.02	0.04	0.06	−0.15^*^	−0.05	**0.16**	−0.08
PAR	**0.11**	(−0.001)	0.15	−0.04	0.11	0.04	0.10	0.06	0.20	0.023	0.02	−0.08
R2	0.95	(0.93)	0.86	(0.73)	0.94	(0.88)	0.88	(0.86)	0.88	(0.84)	0.93	(0.85)

* *p < 0.05*,

** *p < 0.001, p < 0.06*.

More precisely, the maternal psychic status revealed to have a significant influence on the PDMS-2 total motor competence (TMQ) [*F*_(6, 41)_ = 140,337; *p* < 0.001], showing two SCL-90-R symptoms, in addition to age, as predictive factors: somatization and paranoia, with an opposite influence on the TMQ (see Table 5). When mothers tend to experience psychological distress in the form of somatic symptoms, their babies showed a more restrained TMQ. On the contrary, when mothers tend to have paranoiac ideas, their infants show better scores on TMQ.

Considering the PDMS-2 subscales, a higher level of maternal somatization seemed to restrict the infants' natural ability to react [Reflexes: *F*_(6, 41)_ = 41.607; *p* < 0.001]. In addition, maternal depression drove infants to develop better stationary competence [Stationary; *F*_(6, 41)_ = 102.860; *p* < 0.001], while feelings of hostility induced an opposite effect, reducing the ability to reach stationary positions (see Table 5 for Beta values and significance). No influences of psychopathological symptoms arose for infants' locomotion skill, while fine-motor abilities, i.e. grasping and visual-motor integration, were differently affected by maternal hostility thoughts. This mothers' psychological status restricted the grasping actions, which represent activities that require a shared space, not only in physical terms but also from an emotional point of view [Grasping: *F*_(6, 41)_ = 51.824; *p* < 0.001]. Diversely, maternal hostility feelings lead infants to greater visual-motor control [Visual-motor Integration: *F*_(6, 41)_ = 94.106; *p* < 0.001].

## Discussion

None of the mothers involved in this study presented a psychopathological diagnosed condition but they were considered as belonging to two different groups according to the presence/absence of psychological difficulties (according to the reliability of the SCL-90-R questionnaire). Their babies belonged to a normative population as well and showed no developmental issues. Infants' performances at the PDMS-2 were within normal range, based on the Italian reference norms (the Italian version of the test revealed good reliability, Biancotto et al., 2016).

Therefore, the aim of this study was not to verify a causal relation between psychopathological maternal conditions and infants' motor development; rather, our goal was to explore how psychological differences in mothers may influence their approach to the baby and therefore the baby's motor development during the first year of life. For this reason, the sample of mothers was divided into two groups according to their perceived symptomatology (GSI, PST, and PSDI scores of the SCL-90-R questionnaire have been considered as discriminants), in order to distinguish a group of mothers who presented psychological discomfort at a subclinical level and a normative one.

As mentioned above, our mother-infant dyads neither had a diagnosed depression condition nor a delay in motor development. Moreover, even the group of mothers who reported a positive symptomatology for depression, somatization, hostility, paranoid ideation and interpersonal sensitivity symptoms did not affect the general motor development of their infant, because no significant differences were found in the motor abilities between infants who belonged to the two groups of mothers (normative vs. sub-clinical one). This result is in accordance with Cornish et al. (2005), who found that only chronic maternal depression was associated with poorer infant psychomotor development (with the effects being similar for boys and girls), while brief maternal depression did not significantly impact the infant's performance (Cornish et al., 2005). The data we collected also showed no differences related to the gender of the infants (males vs. females), in line with the results of Cornish et al. (2005). Several studies have focused on the effect of depression condition on infants' development during the first year of life and showed the detrimental effects of maternal psychological symptomatology—especially Post Partum Depression- on babies' development (Lyons-Ruth et al., 1986; Galler et al., 2000; Nasreen et al., 2013). Conversely it is not surprising at all that, maternal sub-threshold symptomatology did not affect general motor development.

Once again, our intent was to investigate whether, in a non-clinical sample, any particular relationship could be outlined between maternal symptomatology and the quality of infant motor behaviors. Our study focused on a health safeguarding perspective regarding mother-infant dyads. From this point of view, it is worth identifying whether a certain maternal psychological status can contribute to shape infant motor experiences. It would be interesting to understand if different maternal psychological conditions or feelings can influence the quality of motor behaviors; that is, can infants be directed in their motor experiences by perceiving their mothers' dispositions? The results of the correlation analysis drive us to some considerations. First of all, in the normative sample of our population almost no maternal score correlates with the infants' performances at the PDMS-2; on the contrary, evaluating the sample defined as “subclinical,” several maternal self-perceived symptomatology positively correlates with the infants' PDMS-2 scores. Secondly, infant motor development resulted similar within the two maternal groups, although mothers who reported a psychological discomfort seemed to influence the way in which their babies had motor experiences. Precisely, in the group of normative mothers, a positive correlation was found between the level of interpersonal sensitivity and the infant locomotion competence, while low hostility related to good experiences in the fine-motor domain (see Table 3 for the correlation value between hostility and FMQ score). However, the regression analysis revealed that when mothers perceived overall psychological well-being, their babies' motor development was predicted only by the infant's age. This finding is extremely interesting, because it highlights that when an infant can benefit from a mother who has positive feelings regarding herself and others, he/she lives in the most suitable conditions for his/her motor development. When mothers feel self-confident, emotionally supported and adequate in comparison to other people, infants feel free to explore the environment. After all, emerging action capabilities are crucially shaped by an individual's interactions with the environment: one of the most important motives that drive actions and thus development is social interaction (in addition to exploration). The social motive is expressed from birth by the tendency to fixate social stimuli, imitate basic gestures, and engage in social interaction. The social motive is so important that, apparently without it, a person would stop developing entirely (Stern, 1977; Von Hofsten, 2007). On the contrary, when the maternal levels of symptomatology exceeded the range of normality, they correlated and predicted several infants' PDMS-2 scores. In our sample, a negative correlation was found between maternal somatization symptoms and infant's reflexes behavior, which suggest that, in such maternal status, infant's ability to automatically respond to environmental events are restricted. In other words, the outcome of regression analysis showed that, when mothers reported a higher level of somatization, their infant's motor behaviors were non adjusted to future states in a prospective way. Actually, reflexes are not subject to learning, neither adjusted to meet goals or attain other advantages than those for which they originally emerged, but even so, on the basis of our findings, maternal somatization could lead to a reduction of infant's reflexes. Furthermore, maternal hostility thoughts seemed to produce a reduction of stationary positions and grasping behaviors in infants. It is worthy to note that stationary and grasping behaviors always need adult support. The caregiver usually holds the baby up with her hands to help him/her stand up, and in grasping actions the infant reaches an object mainly to share it with the caregiver within a heartfelt inter-subjective space. Therefore, maternal hostility feelings toward others could limit infant's stationary as well as grasping motor behaviors which require abilities to share.

Considering the positive correlations found and the predictive factors that emerged from regression model analysis, symptoms of depression in subclinical mothers seemed to facilitate the development of stationary position abilities in infants, but this could be due to the infants' attempts to stimulate/activate their mothers who feel depressed and engage them into a relationship. In addition, while hostility feelings seemed to limit stationary and grasping behaviors, the same mental status positively influenced the development of visual-motor integration competences (as assessed by items of the PDMS-2). Therefore, maternal hostility condition seemed to drive infants toward improving their abilities to integrate visual and manual movements. The improvement in visual-motor abilities could not be due to better mastery of the reach-to-grasp action but it could be caused by the infant's need to control what is occurring in the shared space with the caregiver. In fact, each of the items included in the PDMS-2 visual-motor integration subscale requires shared action space, as well as engagement with mothers. Therefore, one could speculate that the more the maternal status is characterized by hostile symptoms, the more infants could be pushed toward massive visual-motor control, instead of acquiring knowledge about themselves, others and their environment though actions or enjoyment of interactions.

Considering the discussed results about reflexes and visual-motor abilities, it is important to consider that infants usually explore objects not only for their own benefit, but also to share their newly acquired knowledge with other people. In their study, Karasik et al. (2011) found that in a large majority of cases, infants showed the objects to the parent at hand and they often carried the objects to them. In this way, social motivation places the infant in the broader context of humans that provide information, comfort, and security. Conversely, when mothers feel depressed or hostile, their infants may react by reaching the stationary position earlier and strongly controlling visually guided actions. In our results, the subclinical maternal group reported hostility feeling that positively correlated with the grasping subscale of the PDMS-2 test. This motor behavior has another very important function for babies. When the hand moves toward an object of interest, it enters the infant's visual field and its movements can then be visually perceived and controlled by visual information. The function of these “built-in” skills is to provide activity-dependent input for the sensory-motor and cognitive systems. This allows the infant to explore the relationship between voluntary commands and movements, between vision and proprioception, and to discover the possibilities and the constraints of his/her actions. In addition, even before birth, the reach-to-grasp action seems to have a social cue, such as the possibility to reach another human being (Castiello et al., 2010). In this case, it can be hypothesized that when depressive symptoms are present, even at a subclinical level, infants try to use their grasping ability to engage their mothers and maintain contact with them.

The influence of maternal psychological status is not confined to fine-motor development; when considering the subclinical group, higher scores of the mothers in the SCL-90-R scales (Somatization, Interpersonal Sensitivity, Depression, Hostility and Paranoia) also tend to influence the performance of infants in the PDMS-2 gross-motor abilities, particularly in the stationary scale. At 6 months of age, infants can sit if supported, rotate their head to look around, bear their weight on their legs and skip energetically when helped to stand, while during the second semester they learn how to sit independently, to crawl and to stand up (Sheridan, 2009). Infants also try to control their posture more and more efficiently. In this prospect, motor development, in terms of movement through space and exploration of the surrounding environment, seems to be positively stimulated by maternal perceived depressive symptomatology. It is possible that mothers with depressive non-clinical symptoms induce their own children to autonomously explore the environment, while baby benefits from new neuronal pathways, improvements in perception or biomechanical changes that allow him/her to explore what surrounding objects and events afford in terms of new action modes (Gibson and Pick, 2000; Adolph and Berger, 2006). However, speculation is that in this way the baby can “stimulate” his/her-own mother in terms of movements, displacements, balance, etc. Moreover, it is intriguing to observe that paranoid ideas seemed to produce a similar effect in pushing infant toward the development of good motor skill. On the other hand, when mothers perceive hostility feelings, the effect on motor development seems to change depending on the specific motor ability: stationary, grasping or visual-motor integration skills.

Drawing from these results, a further step in this work could involve the study of the reciprocal influence in the mother-child dyad during the first 3 years of life or beyond, (with a specific set of reach-to-grasp stationary and locomotion actions) to verify if specific psychological conditions influence motor development in specific directions. Certainly, a wider sample and a longitudinal study could provide information about the long term influence of maternal status. It would also be appropriate to investigate whether the relational space and style developed within the dyad may have consequences not only on the quality of movement, but also on behavioral issues such as hyperactivity and impulsive behaviors).

Considering that the relationship between postpartum depression and outcomes on child development is not linear or direct and that different variables such as genetic influences, mother's education level, child's gender, economic status and father's or other adults' involvement might play a role, (Sohr-Preston and Scaramella, 2006), other factors have been considered in this study. Together with the maternal symptomatic condition and infant motor development, previous experiences of abortion, difficult pregnancies, the level of instruction and the employment status of the mother, previous other children and partnership satisfaction have all been controlled.

These results underline the clinical importance of considering both the maternal status and the development of the infant in the perinatal period, Unfortunately, within the national health service, the attention during pregnancy and the infant's early stages of life is mainly focused on the woman; nobody worries about the infant's condition until he/she reaches the age of three, when some behaviors are already set.

## Author contributions

GP, SB, CF, and CM collected the data for the research. GP, SB, and AS analyzed and interpreted the data. GP, SB, CF, CM, and AS drafted the work. GP, AS, MB, and SZ critically revised the manuscript for important intellectual content and give the final approval of the version to be published. GP and AS agree to be accountable for all aspects of the work in ensuring that questions related to the accuracy or integrity of any part of the work are appropriately investigated and resolved.

### Conflict of interest statement

The authors declare that the research was conducted in the absence of any commercial or financial relationships that could be construed as a potential conflict of interest.
